# High Magnitude Advanced Colorectal Cancer at Diagnosis in Ethiopian Patients: Imaging Pattern and Associated Factors

**DOI:** 10.4314/ejhs.v33i1.11

**Published:** 2023-01

**Authors:** Assefa Getachew Kebede, Tesfaye Kebede, Asfaw Atnafu

**Affiliations:** 1 Department of radiology, School of Medicine, College of Health Sciences, Addis Ababa University

**Keywords:** Colorectal cancer, rectal cancer, stage, diagnosis

## Abstract

**Background:**

Colorectal cancer (CRC) is one of the most prevalent and incident cancers worldwide with an Increasing prevalence in a younger age in developing countries. The aim of the study was to determine the staging and imaging pattern of CRC at diagnosis.

**Methods:**

This is a descriptive cross-sectional study including all consecutive cases of CRC found in the departments of radiology and oncology during the study period from March 2016 - February 2017.

**Results:**

A total of 132 CRC cases were studied with M: F = 2.4:1, mean age of 46yrs and 67.4% </= 50yrs. Left-sided tumors were associated with rectal bleeding (p = 0.001) and bowel habit change (p =0.045) whereas right-side tumors were associated with weight loss (p = 0.02) and abdominal pain (p = 0.004). 84.5% of CRC presented at an advanced stage, and 32% had distant metastasis. Young age was associated with the advanced stage (P=0.006) whereas family history was associated with the lower stage (P=0.008). Distance metastasis was associated with Colonic lesions (P=0.003) and emergent presentation (P=0.008). Asymmetric wall thickening with luminal narrowing was significantly associated with left side tumor (95% vs 21.4%) whereas large mass with necrosis was significantly associated with right side tumor (50% vs 5%) (P= 0.004),

**Conclusions:**

CRC is presented at a younger age and at an advanced stage. The majority of CRCs were left-sided and rectal. Increasing the index of suspicion for CRC in patients with rectal bleeding and, bowel habit change is recommended

## Introduction

Colon and rectal cancer (CRC) is one of the most prevalent and incident cancers worldwide, accounting for 1 in 10 of all cancer cases. Globally CRC is the second cause of cancer death and third in terms of incidence. It also causes more deaths in men than in women worldwide (1, 2).

Knowledge of the location and extent of the primary tumor at initial diagnosis is critical for proper management of the disease as the prognosis of CRC is directly related to the stage at presentation. In UK, approximately 70% of cases involve the colon, and the remaining 30% involve the anus and rectum (3). Similarly, a report from Greece showed a proximal migration of tumors over time (4).

There are scanty reports from Ethiopia regarding CRC. One single report from Israel showed a high prevalence of CRC (19% of all cancers) in Ethiopian Jewish . Few reports from the teaching hospitals in Ethiopia showed younger age and advanced stage at the time of diagnosis (5–8).

Several recent reports from Asia, middle east, and Africa showed an increased incidence of CRC in the younger age group(8–15). The vast majority of CRC diagnoses are symptomatic and even in a population where there is universal coverage of health, only 7% of CRC were detected with screening (16).

The prognosis of CRC is dependent on the stage at the time of diagnosis which is assessed by the depth of wall invasion, and the presence of regional and distant metastasis(5, 8,17,18). Both transrectal ultrasonography and magnetic resonance imaging (MRI) of the pelvis are the modality of choice for locoregional staging while a CT scan of the abdomen and pelvis is recommended for complete staging (19). To the author's knowledge, there is no report regarding imaging patterns and staging of CRC in Ethiopia. Hence, the main objective of this study was to assess the preoperative staging and imaging pattern of CRC at the time of diagnosis in Ethiopian patients

## Material and Methods

**Study area**: The study was conducted in Tikur Anbessa Specialized Hospital (TASH), a tertiary teaching university Hospital which has clinical oncology service , colorectal surgery , pathology, endoscopy and advanced imaging like MRI and MDCT imaging.

**Study design and period**: A cross-sectional descriptive Methodology was employed In TASH during the study period (March 2016 - February -2018).

**Study population**: All patients with CRC 18 years and above who were in TASH at department of radiology, department of oncology or cases brought for multidisciplinary team of gastrointestinal tumor board (GIMDT) during the study period (March 2016 - February -2018).

**Sample size and sampling**: We used a non-probability convenient sampling by taking all study subjects which fulfill the inclusion criteria during the study period (March 2016 - February -2018). Hence ll 132 consecutive subjects with CRC whose radiologic imaging studies available for review were enrolled in the study.

**Data collection and analysis**: Data collected using a structured questionnaire from the medical record, GIT Multi-disciplinary tumor board record, review of patients imaging and interview of the patient when available and necessary. The Medical record of all 132 patients were reviewed for clinical staging, type of work up for the CRC, histopathology diagnosis , treatment outcomes and other relevant information

**Direct interview**: Study subjects were made for 83 patients to collect additional sociodemographic characteristics and clinical information using a structured questionnaire. CT scan of the abdomen and MRI of the pelvis were reviewed by senior radiologist with subspecialty in body imaging.

**Radiologic information include**: tumor location, morphologic appearance, length of tumor and TNM staging (19). **D**ata was entered using Epi info version 3.4 and analyzed using with SPSS version 21 .Descriptive statistics were used to describe findings. The frequency distributions of all variables were checked and percentages, means, median, and proportions were used to describe the findings. Simple 2X2 and binary logistic regression used to look for association between variables; *P*< 0.05 was considered statistically significant.

**The following operational definitions are used**

**Right-sided tumor**: a tumor that involve appendix, ascending colon, hepatic flexure and transverse colon. Left sided tumors: a tumor that involve splenic flexure, descending colon, sigmoid colon and rectum including the anus.

**High rectum**: the part of the rectum between 10cm-15 from the anorectal angle, mid rectum: the part of the rectum between 5cm-10 cm from the anorectal angle, and lower rectum : the segment which is less than 5cm from the anorectal angle.

**CRC**: labeled as advanced when the tumor is in stage III and IV by imaging whereas considered localized tumors when the tumor is at stage I and stage II.

**Young age**: age of 50 years and younger and age above 50 years was considered as older (20).

**Ethical consideration**: Ethical approval was obtained from the ethical review committee of the department of radiology of college of heath of health sciences of Addis Ababa university. Confidentiality of patient information was maintained by avoiding patient identifiers in the data base . Informed consent is obtained from the patient during interview and official permission was obtained to have access to patient record.

## Results

Among the 132 study subjects with diagnosis of CRC enrolled in the study, 70.5% were male and 29.5% were female, with a male to female ratio of 2:1. The mean age of presentation was 46.7years (+/- 14.78yrs), lower for male (46.1 years) than female (48.1years) but the difference was not statically significant. Two thirds of our patients came from the urban and the rest one third were from the rural areas (Table 1).

**Table 1:** Socio-demographic characteristics and Clinical presentation of the study population

Variables		Male	female	Total
**Age group in years**	**</25**	3(3.2%)	3(7.7%)	6(4.5%)
	**26–35**	21(22.6%	4(10.3)	25 (18.9%)
	**36–45**	26(28%)	12(30.8%)	**38(28.8%)**
	**46–55**	18(19.4%)	8(20.5%)	26(19.7%)
	**56–65**	17(18.3%)	8(20.5%)	25(18.9%)
	**> 65**	8(6.1%)	4(3%)	12(9.1%)
	**Total**	93(70.5%)	39(29.5%	132(100%)
**Age group**	**</= 50yrs**	62(69.7%)	27(30.3%)	**89(67.4%)**
	**> 50 years**	31(72.1%)	12(27.9%)	43(32.6%)
**Mean Age (years)**		**46.1(±14.34)**	**48.2(±15.85)**	**46.7(±14.78)**
**Address**	**Urban**	42(77.8%)	12 (22.2%)	54(65%)
	**Rural**	19(65.5%)	10 (34.5%)	29 (35%)
**Occupation**	**Government employee**	23(36.5%)	5(25%)	**28(33.7%)**
	**Farmer**	15(23.8%)	2(10%)	**17((20.5%)**
	**House wife**	5 (7.9%)	11(5%)	16(19.3%)
	**Priv business employee**	9 (14.3%)	0	9(10.8%)
	**Merchant**	5(7.9%)	1(5%)	6(7.2)
	**Daily laborer**	5(7.9%)	1(5%)	6(7.2%)
	**Others**	**1(1.6%)**	**0**	**1(1.2%)**
**Presenting symptoms (count = 88)**				
**Rectal bleeding**		44 (64.7%)	13 (65%)	57 (64.8%)
**Diarrhea**		22 (32.4%)	4 (20%)	26 (29%)
**Constipation**		37(54.4%)	11(55%)	48 (54.5%)
**Tenesmus**		31(45.6%)	9(45%)	40(45.5%)
**Anemia**		8 (11.8%)	3(15%)	11(12.5%)
**Abdominal distension**		16(23.5%)	2(10%)	18(20.5%)
**Fatigue**		28 (41.2%)	9(45%)	37(42%)
**Weight loss**		35 (51.5%)	9(45%)	44(50%)
**Abdominal and pelvic pain**		25(36.8%)	8(40%)	33(37.5%)
**Intestinal obstruction**		12(17.1%)	4(19%)	16(17.6%)

With regards to the presentation, 99.1 % of the study subjects had one or more of the signs and symptoms of CRC and only 0.9 % patients detected incidentally. The commonest form of presentations were rectal bleeding (64.8%), constipation (54.4%) , weight loss (52%), tenesmus (45.6%) and fatigue (42%). Other less frequent symptoms include pelvic and abdominal pain (37.8%), diarrhea (30%), abdominal distension (19%) and anemia (12%). Regarding the mode of presentation, 12 % present acutely with signs of intestinal obstruction but nearly 88% present insidious onset and long duration of symptoms (Table 1). The Mean duration of symptom was 13.9 months; 14.7 months for left sided and 7.3 months for right sided tumors. Compared with rectal cancers, colon cancers most frequently presented with emergency. Regarding some associated factors, sedentary life was observed in 31.3% of patients with CRC, family history of cancer was observed in 12%., frequent intake of red meat in 16.9%, smoking 13.4% and frequent alcohol intake in 6% (all of them were males).

Imaging evaluation was assessed for location of the cancer, morphologic imaging appearance and staging of the tumors; over 2/3 (69.2%) were located in the rectum, 28.2% in the colon and 3 (2.6%) in the anal region. Among the rectal cancers; 82.8% were low rectal, 9.2% were mid and 8% were high rectal cancers. Nearly 57% of all cases of CRCs were low rectal cancer (Table 2). We have got one case of synchronous tumors involving the rectum and transverse colon. Of all cases of CRC, 87.5% were left sided, the rest (12.5%) were right sided tumors and nearly 72% of CRC represent rectal and anal tumors. Left sided tumors were significantly associated with rectal bleeding (P= 0.001) and bowel habit change (P = 0.045) whereas right sided tumors were significantly associated with weight loss (P = 0.02) and pelvic and abdominal pain (P =0.004) (Table 3). Asymmetric wall thickening with luminal narrowing was significantly associated with left side tumor (95% vs 21.4%) where as large mass with necrosis was significantly associated with right side tumors (50% vs 5%) (P= 0.004), morphologic appearance of the CRC was assessed based on CT and MRI appearance; among which majority 89.2% showed annular or semi annular asymmetric wall thickening and smaller proportions present with fungating mass (5.4%) and polypoid mass (4.5%). No significant association was found between morphological appearance with socio-demographic features, staging, or histological features.

**Table 2:** imaging pattern of CRC stratified by sex: CRC in Ethiopian patients imaging pattern and staging at the time of diagnosis

	Variables	*Male*	*Female*	*Total*
**Location of tumor**	Rectum	64(68.8%)	24(61.5%)	88 (66.7%)
	colon	26 (28%)	15 (38.5%)	39 ((31.1%)
	Anal	3(3.2%)	0	3 (2.3%)
**Location in rectum**	Low rectal + anorectal	55 (85.9%)	17 (73.9%)	72 (82.8%)
	Mid rectal	4(6.3%)	4 (17.4%)	8(9.2%)
	High rectal	5 (7.8%)	2 (8.7%)	7((8%)
**Location in colon**	Sigmoid	10(37%)	9(60%)	19(45.2%)
	Descending colon &splenic Flexure	1(3.7%)	3(20%)	4(9.5%)
	Ascending . Colon and Hepatic Flexure	10(37%)	1(6.7%)	11(26.2%)
	Transverse Colon	5(18.5%)	0	5(11.9%)
	cecum	1(3.7%)	2(13.2%)	3(7.1%)
**Morphologic appearance**	Annular/semi annular	70(89.7%)	31(91.2%)	101(90.2%)
	Fungating	6(7.7%)	0	6(5.4%)
	Polypoid	2(2.6%)	3(8.8%)	5(4.5%)
**Staging by imaging**	Stage I	5 (6%)	2(5.9%)	7 (6%)
	Stage II	7 (8.4%)	3 (8.8%)	10(8.5%)
	Stage III	47 (56.6%)	18 (52.9%)	65 (55.6%)
	Stage IV	24 (28.9%)	11(32.4%)	35 (29.9%)
**Histological types**	Adenocarcinoma unspecified	44(74.6%)	17(85%)	61(77.2%)
	Mucinous adenocarcinoma	6(10.2%)	1(5%)	7(8.9%)
	Signet ring adenocarcinoma	4(6.8%)	1(5%)	5(6.3%)
	Squamous cell adenocarcinoma	4(6.8%)	0	4(5.1%)
	Undifferentiated Adenocarcinoma	0	1(5%)	1(1.3%)
	Medullary carcinoma	1(1.7%)	0	1(1.3%)

**Table 3:** Association of clinical features with anatomic location of tumor: CRC in Ethiopian patients imaging pattern and staging at the time of diagnosis, 2017

Clinical presentation	locate tumor		Total n(%)	P-value

	Left colon n(%)	right colon n(%)		
**Rectal bleeding**	**51(71.8%)**	3(21.4%)	54(63.5%)	**0.001**
**Diarrhea**	22(31%)	4(28.6%)	26(30.6%)	
**Constipation**	40(56.3%)	7(50%)	47(55.3%)	
**Tenesmus**	**36(50.7%)**	3(21.4%)	39(45.9%)	**0.045**
**Anemia**	8(11.3%)	3(21.4%)	11(12.9%)	
**Abdominal distension**	13(18.3%)	5(35.7%	18(21.2%)	
**Fatigue**	27(38%)	9(64.2%)	36(42.4%)	
**Weight loss**	32(45.1%)	**11(78.6%)**	43(50.6%)	**0.022**
**Abdominal and pelvic pain**	22(31%)	**10(71.4%)**	32(37.6%)	**0.004**
**Intestinal obstruction**	13(18.3%)	3(21.4%)	16(18.2%)	
**Total**	71(83.5)	14((16.5)	85	
**Duration of symptoms in months** **(mean)**	14.7mon	7.3 month	13.9month	
**Length of tumor (mean) in cm**	8.3cm	9.3cm	8.49cm	
**Mode of presentation**				
**Emergency**	11(10.7%)	3(18.8%)	14(11.8%)	
**Elective**	92(89.3%)	13(81.3%)	105(88.2%	

Radiologic staging at the time of diagnosis; nearly 85% were advanced (stage III-IV) and the rest were localized (stage I-II). Based on TNM staging 32.4% of cases presented with distant metastasis, 77% showed Lymph node metastasis, and 88.8% were T3 and T4. Staging of CRC was significantly associated with age and family history. Being younger age, (age </= 50yrs) was significantly associated with advanced staging (p = 0.006) and those patients with a family history of cancer showed relatively localized disease than those without family history (p= 0.008) (Table 4). Though the overall staging was not associated with the location and morphological appearance, distant metastasis was significantly associated with colonic tumors (0.03, chi = 11.3) and those presenting with an emergency (P = 0.008) (Table 5).

**Table 4:** Frequency distribution of factors by stage of CRC and sex: CRC in Ethiopian patients imaging pattern and staging at the time of diagnosis

Associated factors		Localized	Advanced	p value	Male	Female
					
		No(%)	No(%)			
**Alcohol **	None	6(17.1)	29(82.)		25(39.7%)	16(60%)
	Infrequent	6(17.6)	28 (82.4)		33 (52.4%)	4 (20%)
	Frequent	0	4(100)		5(7.9%)	0
**Daily**	Sedentary	1(4.8)	20(95.2)		16(25.4%)	10(50%)
**physical**	frequent	6(25)	18(75)		21(33.3%)	4(20%)
**activity**	vigorous	5(17.9)	23(82.1)		26(41.3)	6(30%)
**Sex**	Male	12(14.5)	71(85.5)			
	female	6(17.6)	28(82.4)			
**Age**	</= 50yr	7(38.9%)	73(73,7%)	0.006	62(69.7%)	27(30.3%)
	> 50yr	11(61.1%)	6(26.3%		31(72.1%)	12(27.9%)
**Family History**	No	7(11.1)	56(88.9)		55(87.3%)	18(90%)
	Yes	5(50%)	5(50%)	0.008	8(12.7%)	2(10%)
**Self-History of polyp or cancer**	No	12(16.9)	59(83.1)		61(96.8%)	20(100%)
	Yes	0	2		2(3.2%)	0
**Intake of red meat**	none	0	3		4(6.3%)	0
	Infrequent	10(17.5)	47(82.5)		48(76.2%)	17(85%)
	frequent	2(15.4)	11(84.6)		11(17.5%)	3(15%)

**Table 5:** Distribution of the various factors stratified by distant metastasis: CRC in Ethiopian patients imaging pattern and staging at the time of diagnosis

		Metastasis		Total	p-value
				
		M0	M1		
**mode of**	Elective	65(73.9%)	23(26.1%)	88 (88%)	
**presentation**	emergency	4(33.3%)	**8(66.7%)**	12(12%)	**P = 0.008**
**Family History**	Yes	8(80%)	2(20%)	10(14.7%)	
	No	41(70.7%)	17(29.3%)	58(85.3%)	
**Age group 50**	</= 50	47(64.4%)	26 (35.6%)	73 (67.6%)	
	> 50	26(74.3)	9(25.7%)	35(32.4%)	
**Location of tumor**	rectum	55(76.4%)	17(23.6%)	72 (66.7%)	**P= 003**
	colon	15(45.5%)	**18(54.5%)**	33(30.6%)	
	anal	3	0	3(2.8%)	
**Imaging feature**	Annular/semi-annular	15(62.5%)	9(37.5%)	24(24.5%)	
	Asymmetric thickening with stricture	43(72.9%)	16(27.1%)	59(60.2%)	
	large mass with necrosis	6(54.5%)	5(45.5%)	11(11.2%)	
	Polypoid Intra luminal	3(75%)	1(25%)	4(4.1%)	
**Sex**	Male	52(68.4%)	24(31.6%)	76	
	Female	21(63.6%)	11(36.4%)	33	
**Histology**	Adeno CA	43(76.8%)	13(23.2%)	56	
	Mucinous Adeno	4(66.7%)	2(33.3%)	6	
	SRCCA	3(60%)	2(40%)	5	
	SCCA + medullary	4(75%)	1(25%)	5	

The histological type of CRC: among the study subjects, 77.2% of the CRC were conventional Adenocarcinomas (in which no sub-type was specified), 8.9% were Mucinous Adenocarcinoma, 6.3% signet ring cell adenocarcinoma (SRCC) and 5.1% were Squamous cell carcinoma (SCC). all of the SCC was in anal and anorectal locations. Four out of five SRCCs were in the rectum and out of which three were in the low rectal region. There was no significant difference in the staging of the tumor compared to the histological type but all SRCCs were in advanced stages. All SRCCs occurred in age 50 years or younger with a mean age of 35yrs whereas SCC found above the 6th decade. Four out of five SRCCs were males.

## Discussion

In this study, CRC presented at younger age and at an advanced stage at the time of diagnosis with long durations of symptoms before the diagnosis. The majority of the CRCs in our study were located on the left side and were predominantly rectal cancer.

One of the key findings in this study is the predominance of young patients with CRC with a mean age of 46.7 years and 67.4% of which were < 50 years of age. This finding is contrary to the western world in which the mean age of presentation was reported to be 67- 69yrs and only 1–9% of them were < 40 years. Whereas, our finding is in line with previous reports from the same institution (46–47 years) (8,9), reports from various African (46–56years) (21,22), Asian and middle Eastern countries (41–58 years) (23–27). We have also noted a marked male predominantly of CRC (2.4: 1) which has a similar pattern but is higher than global and African estimates of 1.5:1 and 1.2–1.6:1 respectively (27). This male predominance was marked in CRC occurring in young while an increasing proportion of females seen in older ages could be explained by a speculated protective effect of estrogen (4).

In this study, nearly 89% of cases of CRC were locality advanced and 32% had distant metastasis at diagnosis. The findings of the high magnitude of the advanced stage at diagnosis are in agreement with many studies around the world (5,21,28,29–30). The advanced stage at presentation was significantly associated with younger age which is also in agreement with many other studies demonstrating an aggressive feature and an advanced stage of CRC in a young (31,32). Whereas the family history of cancer was significantly associated with more localized disease (P=0.008) which could be because those with a family history tend to have a better awareness and are likely to get medical attention and be screened for cancer earlier than those without.

Regarding presenting symptoms, our study demonstrated that 99.1 % of cases presented with one or more symptoms with a mean duration of the symptom of 13.4 months which is not surprising as there is no practice of active surveillance of CRC in at-risk populations in our country. even In one study done in Canada, a country where there is more prevalence of CRC, active surveillance detected only 7 % of CRC (16), suggesting that a significant majority present and diagnosed with symptoms even in the developed nation. According to a report by graham et .al the presence of symptoms at presentation is associated with a worse prognosis (28). The long duration of symptoms and delay in diagnosis is the likely contributing factor for the advanced stage of presentation in our study population.

The long duration of symptoms in our study population (13.4 months) is comparable to studies done in Nigeria (14.5months), and Tanzania (22 months), but higher than a report by O'Connell et al (6.2months), Graham et l (6 months) and India 4 months (*16,21,30,31).* Distant metastasis was noted in 32.4% of our cases at the time of diagnosis which is comparable to study findings from Africa and Asia (24-36%) but higher than reports from the west (18%) (*5,16, 21,29).* Distant metastasis was significantly associated with colonic tumors (P=0.003) compared with rectal CA and those presented with an emergency (p= 0.008) Which could be due to the subtle presentation of colonic tumors at an early stage and because of which it may present with complications like intestinal obstruction and detected at an advanced stage.

The sub-site distribution of CRC in our study demonstrated that 87.5% were left-sided tumors and nearly 69% were rectal, which is contrary to the sub-site distribution pattern seen in the western world (33**)**, whereas our findings are in line with many reports from Africa and other developing nations documenting a predominantly distal tumor which is significant in young-onset CRC (13,16,20,27).

As seen in our study various Studies on CRC in young adults have produced several consistent findings, most notable of which are presenting in an advanced stage, location in the distal colon and rectum, and associated aggressive pathologic features like poor differentiation, and Mucinous and signet ring histology.

The sample size was small and some of the findings were too small to draw any statistical significance. As the study was conducted in the teaching referral hospital where there is the only one oncology center, more advanced cases might have been included.

In conclusion, this study demonstrated nearly 85 % of the CRC presented at an advanced stage, in a younger age with male predominance, and a third of them presented with distant metastasis. We have also noted a long mean duration of symptoms before diagnosis (13.4month). Left-side tumor account for 87.5% of CRCs, with predominant symptoms of rectal bleeding and bowel habit change, and imaging appearance of asymmetric thickening and stricture on imaging, whereas right-side tumorassociated weight loss and abdominal pain and present with a large mass with necrosis on imaging.

We recommend creating awareness of CRC in the population and health care providers for early detection of CRC. Also, create a high index of suspicions of CRC in clinicians, especially in young patients presenting with rectal bleeding, bowel habit change, and weight loss. A further multicenter, population-based, and experimental study is recommended to substantial these findings, identify risk factors in a young and develop an appropriate protocol for screening CRC.

## References

[1] Bray F, Ferlay J, Soerjomataram I, Siegel RL, Torre LA, Jemal A (2018). Global cancer statistics 2018: GLOBOCAN estimates of incidence and mortality worldwide for 36 cancers in 185 countries. CA Cancer J Clin.

[2] Jemal A, Bray F, Center MM, Ferlay J, Ward E, Forman D (2011). Global cancer statistics. CA: a cancer journal for clinicians.

[3] Maddams J, Brewster D, Gavin A, Steward J, Elliott J, Utley M (2009). Cancer prevalence in the United Kingdom: estimates for 2008. British journal of cancer.

[4] Efremidou EI, Liratzopoulos N, Papageorgiou SM, Romanidis K, Tourlis T, Kouklakis G (2008). Colorectal carcinoma: correlation between age, gender and subsite distribution. Chirurgia.

[5] Ashenafi S (2000). The frequency of large bowel cancer as seen in Addis Ababa University, Pathology Department. Ethiopian medical journal.

[6] Zemenfes D, Kotisso B (2015). A Two-year review of Colorectal Cancer at Tikur Anbessa Specialized Hospital, Addis Ababa, Ethiopia. East and Central African Journal of Surgery.

[7] Yarom N, Gorbatsevich E, Geva N, Evron E, Kovel S, Cyjon A (2015). Colon cancer in immigrants from Ethiopia. Journal of Clinical Oncology.

[8] Bishehsari F, Mahdavinia M, Vacca M, Malekzadeh R, Mariani-Costantini R (2014). Epidemiological transition of colorectal cancer in developing countries: environmental factors, molecular pathways, and opportunities for prevention. World J Gastroenterol.

[9] Agrawal S, Bhupinderjit A, Bhutani MS, Boardman L, Nguyen C, Romero Y (2005). Colorectal cancer in African Americans. The American journal of gastroenterology.

[10] Al-Ahwal MS, Abdo Al-Ghamdi A (2005). Pattern of colorectal cancer at two hospitals in the western region of Saudi Arabia. Saudi journal of gastroenterology.

[11] Johnson CM, Wei C, Ensor JE, Smolenski DJ, Amos CI, Levin B (2013). Meta-analyses of colorectal cancer risk factors. Cancer causes & control: CCC.

[12] Lee PY, Fletcher WS, Sullivan ES, Vetto JT (1994). Colorectal cancer in young patients: characteristics and outcome. The American surgeon.

[13] Pahlavan PS, Kanthan R (2006). The epidemiology and clinical findings of colorectal cancer in Iran. Journal of gastrointestinal and liver diseases: JGLD.

[14] Pal M (2006). Proportionate increase in incidence of colorectal cancer at an age below 40 years: an observation. Journal of cancer research and therapeutics.

[15] Taha MO, Abdalla AA, Mohamed RS (2015). Pattern & presentation of colorectal cancer in central Sudan, a retrospective descriptive study, 2010-2012. African health sciences.

[16] Smiljanic SR, Gill S (2006). Patterns of diagnosis for colorectal cancer (CRC): Screening detected versus symptomatic presentation. Journal of Clinical Oncology.

[17] Balthazar EJ (1988). Carcinoma of the colon: detection and preoperative staging by CT. AJR.

[18] Gazelle GS, Gaa J, Saini S, Shellito P (1995). Staging of colon carcinoma using water enema CT. Journal of computer assisted tomography.

[19] Gunderson LL, Jessup JM, Sargent DJ, Greene FL, Stewart AK, Revised TN (2010). categorization for colon cancer based on national survival outcomes data. J Clin Oncol.

[20] Dozois EJ, Boardman LA, Suwanthanma W (2008). Young-onset colorectal cancer in patients with no known genetic predisposition: can we increase early recognition and improve outcome?. Medicine (Baltimore).

[21] Chalya PL, McHembe MD, Mabula JB, Rambau PF, Jaka H, Koy M (2013). Clinicopathological patterns and challenges of management of colorectal cancer in a resource-limited setting: a Tanzanian experience. World journal of surgical oncology.

[22] Abudu EK, Akinbami OS (2016). Colorectal Carcinomas in Uyo City, Southern Geopolitical Zone of Nigeria: A Review of Clinicopathological Characteristics and Literature. Rare tumors.

[23] Bhurgri Y, Khan T, Kayani N, Ahmad R, Usman A, Bhurgri A (2011). Incidence and current trends of colorectal malignancies in an unscreened, low risk Pakistan population. Asian Pacific journal of cancer prevention: APJCP.

[24] Amini AQ, Samo KA, Memon AS (2013). Colorectal cancer in younger population: our experience. JPMA The Journal of the Pakistan Medical Association.

[25] Abou-Zeid AA, Khafagy W, Marzouk DM, Alaa A, Mostafa I, Ela MA (2002). Colorectal cancer in Egypt. Dis Colon Rectum.

[26] Li XF, Shen RJ, Liu PL, Tang ZC, He YK (2000). Molecular characters and morphological genetics of CAL gene in Chinese cabbage. Cell research.

[27] Innocent L, Raj T, Sandie Jonathan S (2017). The shifting epidemiology of colorectal cancer in sub-Saharan Africa. Lancet Gastroenterol Hepatol.

[28] Graham A, Adeloye D, Grant L J (2012). Estimating the incidence of colorectal cancer in Sub-Saharan Africa: a systematic analysis. Journal of global health.

[29] Aljebreen AM (2007). Clinico-pathological patterns of colorectal cancer in Saudi Arabia: younger with an advanced stage presentation. Saudi journal of gastroenterology: official journal of the Saudi Gastroenterology Association.

[30] Yawe KT, Bakari AA, Pindiga UH, Mayun AA J (2007). Clinicopathological pattern and challenges in the management of colorectal cancer in Sub-Saharan Africa. Clin Med.

[31] O'Connell JB, Maggard MA, Livingston EH, Yo CK (2004). Colorectal cancer in the young. Am J Surg.

[32] Campos FG (2017). Colorectal cancer in young adults: A difficult challenge. World journal of gastroenterology.

[33] Gatta G, Ciccolallo L, Capocaccia R, Coleman MP, Hakulinen T, Moller H (2003). Differences in colorectal cancer survival between European and US populations: the importance of sub-site and morphology. European journal of cancer.

